# Protective effect of topical *Cordia verbenacea* in a rat periodontitis model: immune-inflammatory, antibacterial and morphometric assays

**DOI:** 10.1186/1472-6882-12-224

**Published:** 2012-11-21

**Authors:** Suzana Peres Pimentel, Guilherme Emerson Barrella, Renato Corrêa Viana Casarin, Fabiano Ribeiro Cirano, Márcio Zaffalon Casati, Mary Ann Foglio, Glyn Mara Figueira, Fernanda Vieira Ribeiro

**Affiliations:** 1 Depto de Odontologia, Universidade Paulista – UNIP, Av. Dr. Bacelar, 1212, 4 andar, Vila Clementino, São Paulo, SP, 04026-002, Brazil; 2 Multidisciplinary Center for Chemical, Biological and Agricultural Research (CPQBA), Campinas University, Campinas, São Paulo, Brazil

**Keywords:** *Cordia Verbenaceae*, Essential oil, Periodontitis, Alveolar bone loss, Anti-inflammatory, Antibacterial

## Abstract

**Background:**

This study evaluated the effects of *C. verbenacea* essential oil topically administered in a rat periodontitis model.

**Methods:**

Periodontitis was induced on rats in one of the mandibular first molars assigned to receive a ligature. Animals were randomly divided into two groups: a) non-treatment group (NT) (n = 18): animals received 1mL of vehicle; b) *C. verbenacea* group (C.v.) (n = 18): animals received 5mg/Kg of essential oils isolated from *C. verbenacea.* The therapies were administered topically 3 times daily for 11 days. Then, the specimens were processed for morphometric analysis of bone loss. The ligatures were used for microbiological assessment of the presence of *Aggregatibacter actinomycetemcomitans*, *Tannerella forsythia* and *Porphyromonas gingivalis* using PCR. The gingival tissue was collected to Elisa assay of interleukin (IL)-1α and IL-10 levels.

**Results:**

Bone loss was inhibited by *C. verbenacea* when compared to the NT group (*p* < 0.05). A decrease in the levels of IL-1α and increase in the IL-10 amounts was observed in the C.v. group as compared to NT group (*p* < 0.05). A lower frequency of *P. gingivalis* was found in C.v. group (*p* < 0.05).

**Conclusion:**

*C. verbenacea* essential oil topically administered diminished alveolar bone resorption, promoting a positive local imbalance in the pro/anti-inflammatory system and reducing the frequency of detection of *P. gingivalis*.

## Background

Periodontitis is one of the main chronic inflammatory diseases presenting the most prevalent form of bone pathology in humans [1]. Indeed, a majority of adults suffer from moderate periodontitis, with up to 15% of the population being affected by severe generalized periodontitis at some period in their lives [2]. This disease is characterized by an infectious condition leading to the occurrence of supporting tissue destruction, which is host-mediated by local production of immune-inflammatory markers in response to pathogens and their products [3]. Innumerous pro- and anti-inflammatory mediators have been identified in the gingival tissue or in crevicular fluid as a result of cellular responses to periodontopathogens [4-6]. Among the host mediators produced after microbial recognition, the pro-inflammatory cytokine interleukin (IL)-1 has been described to represent important role in periodontitis pathogenesis, being associated with inflammatory cell migration and osteoclastogenesis progression [7,8]. Conversely to the destructive mechanism that involves pro-inflammatory cytokines, regulatory pathways mediated by anti-inflammatory mediators such as IL-10 can protect periodontal tissues. In fact, it has been evidenced that IL-10(-/-) mice present increased susceptibility to *P. gingivalis*-induced alveolar bone loss, suggesting a role for IL-10 in the control of destructive inflammation [9].

Within this context, studies have investigated strategies to modulate the host’s immune-inflammatory response associated with periodontal disease. Some previous pre-clinical and clinical studies showed that both conventional Nonsteroidal anti-inflammatory drugs (NSAIDs) and those selective inhibitors of Cyclooxygenase-2 (COX-2) are able to modulate a host’s immune-inflammatory reaction [10-15]. However, the systemic use of these drugs is commonly associated with side effects, impairing patients’ compliance to their consumption [16]. Although the local utilization of NSAIDs and other anti-inflammatory agents has been examined in some studies, many of them have revealed unsatisfactory results in controlling inflammation when these drugs are topically administrated [17-19].

To overcome this aspect, there is compelling evidence that many plants or their active components used in traditional medicine might be useful for the treatment of inflammatory conditions [20-22]. Considering the growing interest in plant-derived drugs, studies have focused in the use of natural products to prevent oral diseases such as periodontitis [21-26]. *Cordia verbenacea*, classified as *C. curassavica* and popularly known as Erva Baleeira, is a perennial plant widespread in the Atlantic Forest and distributed along the Brazilian coastal regions. The phytochemical analysis of products obtained from its leaves has revealed several important constituents such as flavonoids, *trans*-caryophyllene, α-humulene and others, displaying marked anti-rheumatic, anti-inflammatory, analgesic, and healing activities [27,28]. The anti-inflammatory effects of *C. verbenacea* or its essential oil have been successfully evidenced in classical models of inflammation and associated with an important protective effect on the gastric mucosa, as well as very low toxicity [27-30]. Besides the anti-inflammatory properties of *C. verbenacea*, studies have evidenced that this medicinal plant also displays an important antimicrobial activity [31-33]. Nevertheless, to date, no study has investigated the impact of *C. verbenacea* in modulating host immune-inflammatory response and in controlling putative periodontal bacteria in the presence of periodontitis.

New evidence regarding the immune-inflammatory action and antibacterial impact of the *C. verbenacea* may provide a wider therapeutic window for subjects with periodontitis. Thus, the aim of present study was to determine the effect of *C. verbenacea* essential oil locally administered in a rat periodontitis model, by evaluating the alveolar bone loss, cytokine levels modulation, and microbiological alterations. The hypothesis was that topical use of *C. verbenacea* could control the pathogens associated with periodontal destruction, and modulate the pattern of immune-inflammatory markers in tissues presenting periodontitis. It could prevent, in consequence, the progression of periodontal disease, representing a promising new approach for the management of periodontitis.

## Methods

### Plant material and extraction of essential oil

Fresh leaves and stems of *C. verbenacea* were collected from the Multidisciplinary Center for Chemical, Biological, and Agricultural Research (CPQBA) of Campinas University (UNICAMP). A voucher specimen (UEC 112744) is deposited at the Biological Institute of UNICAMP. The essential oil was extracted from fresh, chopped leaves by hydro distillation for 4 h, using a Clevenger-type apparatus. Under these conditions, the yield of essential oil was 0.37% (considering 80% humidity). Both the crude ethanol extract and volatile oil were analyzed by GC/MS (HP 6890/mass detector HP 5975/automatic injector 7673 Agilent Technologies, Palo, CA) using a HP-5 fused silica capillary column (30 m × 0.25 mm × 0.25 μm/stationary phase 5% methyl silicone). Helium was used as the carrier gas (1.0 mL/min^-1^). The detector was acquired by electron impact (scan mode) using an ionization energy of 70 eV. One micro liter of sample was injected in the split less mode. The column was initially heated at 60°C and then heated at 3°C/min^-1^ to 240°C. Injector and detector temperatures were 220°C and 250°C, respectively. The compounds were identified by comparing their mass spectra with the system data bank NIST-2005. A homologous series of n-hydrocarbons C-9-C18 and C-20 was co-injected with the sample in order to calculate the retention index and co-injection of authentic standards to provide additional criteria for identification. The essential oil components were therefore identified crossing their retention index, with comparison of their mass spectrums compared to those of authentic samples.

For the quantitative determination of α-humulene, calibration was carried out using a standard solution of α-humulene in acetone (25–127 μg/ml) containing dibutylphthalate (200 μg/ml) as the internal standard. The correlation between the peak area ratio and the concentrations of the compound was linear over the range tested. In order to determine the contents of α-humulene, oil samples (100.00±0.1 mg) were dissolved in acetone (10 ml) containing the internal standard (200 μg/ml) and aliquots (1.0 μl) injected into the GC/MS. All chemical analyses were performed in triplicate. Purity obtained was 99% to α-humulene.

### Rat periodontitis model

The animal cohort was composed of 36 male Wistar rats weighing 308±35 g at the beginning of the study, obtained from the Butantan Institute (São Paulo, Brazil). The rats were 90 d old and were kept in temperature-controlled cages, exposed to a 24-h light–dark cycle of equal time, and had free access to water and food ad libitum (Labina, Purina1, Paulinia, SP, Brazil) in the Bioterium of Paulista University. The experimental procedure was approved by the Paulista University Institutional Animal Care and Use Committee (036/10 CEP/ICS/UNIP).

In order to induce experimental periodontitis, one of the mandibular first molars of each animal was randomly assigned to receive a cotton ligature (Corrente Algodão no. 10; Coats Corrente, São Paulo, SP, Brazil) in a cervical position being knotted submarginally. The ligatures were kept in position in order to allow biofilm accumulation over 11 days [15,22,25]. The contralateral tooth was left unligated to be used as a control. This procedure was performed under general anesthesia by intramuscular administration of ketamine hydrochloride (10 mg/kg) (Dopalen®, Agribrands Brasil Ltda., Paulínia, SP, Brazil) and xylazine hydrochloride (10 mg/kg) (Rompun®, Bayer S.A., São Paulo, SP, Brazil).

### Treatment

After ligature placement, animals were randomly assigned to one of the following groups, according to a computer-generated code: Non-treatment group (NT) (n = 18) animals received topically 1 mL of vehicle and *C. verbenacea* essential oil group (C.v.) (n = 18) animals received topically 5 mg/Kg body wt. of essential oils isolated from *C. verbenacea*. Treatments were topically administered with a 1 mL syringe, three times daily (7 a.m., 1 p.m., and 8 p.m.) for 11 days. The animals were adequately contained by the researchers to carry out the treatments without the need for anesthesia.

The animals were evaluated at each of these moments (7 a.m., 1 p.m., and 8 p.m.) throughout the experiment to assess possible clinical or toxicological symptoms. At the conclusion of the experiment, the animals' weights were monitored and compared to the baseline. The animals were euthanized by CO2 inhalation on the 12th day of periodontitis induction. Subsequently, the mandibles were excised for morphometric analysis. For microbiological assessment, the ligatures were removed using a dental nipper. The buccal gingival tissue from the area surrounding the lower first molar submitted to experimental periodontitis was also collected to enzyme-linked immunosorbent assay (ELISA).

### Measurement of alveolar bone loss

After gingival dissection, the mandibles de-fleshed after immersion in 8% sodium hypochlorite for 4 h. The specimens were washed in running water and immediately dried with compressed air. To distinguish the cementum enamel junction (CEJ), 1% aqueous methylene blue solution (Sigma-Aldrich®, Saint Louis, MO, USA) was applied to the specimens for 1 min and then washed in running water. Photographs were obtained with a 6.1-megapixel digital camera (Canon® 40D, NY, USA) on a tripod to keep the camera parallel to the ground at the minimal focal distance. The specimens were fixed in wax with their occlusal planes parallel to the ground and long axes perpendicular to the camera. Photographs of the buccal aspects were made both in test and control sides. To validate measurement conversions, a millimeter ruler was photographed with all specimens [34]. Alveolar bone loss was determined on the buccal surface of the lower first molars by the distance of the CEJ from the alveolar bone crest, measured at three equally distant sites. The average alveolar bone height was calculated for each tooth.

A single examiner (G.E.B.), who was not aware of the experimental data, carried out morphometric measurements of alveolar bone loss. The measurements were performed after intraexaminer calibration by evaluating 10 non-study images presenting alveolar bone loss similar to the present study. The examiner measured the linear measurements of all photographs twice within 24 hours. The intraclass correlation showed 94.8% reproducibility.

### ELISA assay

The collected tissues were placed into sterile tubes containing 400 μl phosphate-buffered saline (PBS) with 0.05% Tween-20. All samples were stored at -20°C. After, the tissue was weighed, then cut into small pieces (1–2 mm^3^) using scissors, and solubilized in PBS to a final concentration of 100 mg tissue/ml. After extraction on a Vortex mixer for 10 min, each sample was centrifuged at 370 g for 5 min, and the supernatant was collected, divided into small portions, and stored at -70°C until use. To avoid protease activity, the entire procedure was carried out at 4°C. The levels of IL-1α and IL-10 were determined by ELISA using commercially available kits (Quantikine; R&D Systems Inc., MN, USA), according to the manufacturer’s instructions. Samples were diluted with the kits’ diluents and dilution was taken into consideration for the calculation of the concentration of each substance. This concentration was calculated with a standard curve, prepared using the standard proteins in the kit. The standard curve range used for IL-1α measurement was 15.6–1000 pg/ml and for IL-10 the range was 31.2–1000 pg/ml. ELISAs were run in duplicate and mean values were used to calculate concentrations of each marker.

### Microbiological assessment

For detection of *P. gingivalis*, *T. forsythia* and *A. actinomycetemcomitans*, the polymerase chain reaction (PCR) technique was applied using specific primers reported in the literature [35].

Initially, each removed ligature was individually placed in microtubes containing 0.01 M Tris–EDTA solution, pH 8 (TE). The DNA was then extracted from the subgingival biofilm, as previously described [36] The PCR amplification was performed using 5uL sample added to 45uL reaction buffer containing 1.5 mM MgSO_4_, 200 uM deoxynucleotide triphosphate, 2 uM of each primer, and 2U of Taq polymerase enzyme (OneTaq Polymerase, New England Biolabs, Ipswich, MA, USA). Positive and negative controls were used in each run. For reaction, the temperature settings were: for *A. actinomycetemcomitans* and *T. forsythia* detection, an initial denaturation step of 2 min at 95°C, followed by 36 cycles of denaturation and extension at 95°C for 30 seconds (s), annealing at 60°C for 60 s and extension at 68°C for 60 s, and a final elongation step at 68°C for 120 s; for *P. gingivalis* detection, an initial denaturation step at 95°C for 2 min, followed by 32 cycles of denaturation and extension at 95°C for 60 s, annealing at 60°C for 60 s and extension at 68°C for 60 s, and a final elongation step at 68°C for 120 s. Reactions were done (Eppendorf Mastercycler gradient, Hamburg, Germany) and PCR products were separated by electrophoresis in 2% agarose gels and Tris-borate-EDTA running buffer (pH 8.0). The DNA was stained with 0.5 mg of ethidium bromide/mL and visualized under UV illumination. Standardized photographs of the images were taken (Canon EOS 400D, Lake Success, NY, USA) and analyzed. The analyses were performed at the Laboratory of Research of Paulista University by a blinded subject.

### Statistical analyses

To test the null hypothesis that essential oil of *C. verbenacea* had no influence on alveolar bone loss and on cytokine levels, intergroup analysis was performed by student’s t-test. In addition, the paired student’s t-test was used for intragroup comparisons between ligated and unligated teeth. To test the null hypothesis that essential oil of *C. verbenacea* had no effect on periodontal bacteria, intergroup analysis was performed using Fisher’s exact test. The significance level established for all analyses was 5%.

## Results

The animals did not show any signs of systemic illness throughout the study period. The rats also did not lose weight throughout the experimental period. Indeed, the tested therapies did not promote side effects or alterations in the animals’ behavior and in their general activity related to the toxicity. Deaths were not observed.

### Morphometrical results

**Figure 1:**
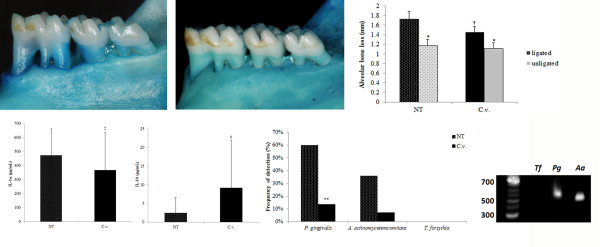
**Panel illustrating data for morphometric, immune-inflammatory, and antibacterial assays.A** and **B**) Representative photographs illustrating the morphometric findings of *C. verbenacea* essential oil group (**A**) and non-treatment group (B). **C**) Means and standard error of the mean (SEM) of alveolar bone loss (mm) for ligated and unligated teeth in both groups. Significant difference when compared to ligated teeth (*). Significant difference when compared to NT group (†) (student t test, *p* < 0.05). **D**) Means and SEM of IL-1α and IL-10 concentration (pg/ml) assayed by ELISA. Significant difference when compared to NT group (‡) by student t test, *p* < 0.05. **E**) Frequency (%) of detection of periodontal pathogens in both groups. Significant difference when compared to NT group (**) by Fisher’s exact test, *p* < 0.05. **F**) Representative reverse transcription–polymerase chain reaction products illustrating periodontal pathogens observed in one animal of the *C. verbenacea* essential oil group.

A significant difference in the alveolar bone loss between unligated and ligated teeth was observed for both groups (*p* < 0.05), showing that the ligatures around the teeth were able to promote bone loss. Measurements of alveolar bone loss in ligated mandibular molars revealed significantly higher bone-loss values in the NT group compared with C.v. group (*p* < 0.05). Figure 1A,C illustrate the morphometric findings.

### Gingival tissue cytokine levels

Figure 1D shows the gingival tissue mediator levels of IL-1α and IL-10 evaluated for both groups. Eleven days after the experimental periodontitis induction, the levels of IL-1α were lower in C.v. group when compared to NT group (*p* < 0.05). Conversely, the levels of IL-10 were higher in tissues treated with *C. verbenacea* essential oil than those not treated (*p* < 0.05).

### Microbiological outcomes

The frequency of detection of the pathogens evaluated in the ligature of each group is presented in Figure 1E. Figure 1F illustrates representatives reverse transcription–PCR products of periodontal pathogens observed in one animal of *C. verbenacea* essential oil group. *T. forsythia* was not detected in any ligature biofilm in both groups. In the C.v. group, *A. actinomycetemcomitans* was found in 7.1% of the rats, whereas in the NT group this pathogen was detected in 36% of the animals. However, no significant difference in the frequency of detection between groups was observed for this pathogen (*p* > 0.05). Regarding *P. gingivalis* detection, the C.v. group presented a statistically lower frequency of detection, 13.5%, than NT group, in which *P. gingivalis* was detected in 60% of ligatures (*p* < 0.05).

## Discussion

Although it is well established that periodontitis is an infectious disease, the host immune and inflammatory response to the microbial challenge has an essential role in the periodontal breakdown [3]. Considering the better understanding of the participation of host immune-inflammatory mediators in disease progression, the use of modulating agents as an adjunctive therapy to the periodontal treatment has been encouraged to provide innovative visions of the management of periodontitis [5,21,22,37,38]. In this context, the use of *C. verbenacea,* a medicinal plant with anti-inflammatory and antimicrobial proprieties, could represent a promising future strategy in treating periodontal diseases. This study evaluated, for the first time, the anti-inflammatory and antibacterial impact of topically administrated *C. verbenacea* in preventing the progression of periodontitis. In general, the outcomes demonstrated that the use of *C. verbenacea* was effective against periodontal pathogens; it also positively modulated immune-inflammatory response, minimizing the alveolar bone loss in ligature-induced periodontitis.

In the current investigation, morphometric analysis demonstrated that the therapy with topical *C. verbenacea* essential oil promoted an important decrease in bone loss, whereas in the non-treated group the periodontal breakdown was higher. This protective effect displayed by *C. verbenacea* treatment may be associated with modulation of the inflammatory reaction, as previously indicated by other research, which revealed that compounds isolated from *C. verbenacea* exerted important anti-inflammatory activity in different experiments [27,29]. In line with these data, Sértie et al. [30] demonstrated that the topical administration of similar doses of *C. verbenacea* than those used in the current investigation significantly inhibited nystatin-induced edema. The marked influence of *C. verbenacea* and its components in attenuating inflammatory disorders could be explained, at least in part, by the downregulation of pro-inflammatory mediators, such as TNF-α and IL-1β [27,39]. In fact, our results demonstrated a reduction in the pro-inflammatory IL-1α levels when the therapy with *C. verbenacea* was applied. The biologic activity of IL-1 is extremely diverse, with the focus on the activation of acute phase proteins, prostaglandins, and other cytokines, the induction of collagen and collagenase synthesis, and calcium resorption in bones [7,8].

Conversely, the topical use of *C. verbenacea* essential oil in this investigation promoted an elevation in the levels of anti-inflammatory IL-10 11 days after the therapy. Of interest, the effects mediated by IL-10 may involve the downregulation of pro-inflammatory markers or also present a protective role in tissue destruction, regulating both matrix metalloproteinases and receptor activator of NF-_К_B (RANK) systems [40,41]. Recently, it has also been suggested that IL-10 has a direct role in bone tissue formation, since the alveolar bone loss in the absence of IL-10 is associated with a reduced expression of osteoblast and osteocyte markers, independently of microbial, inflammatory, or bone-resorptive pathways [42].

In addition to modulating the IL-1 and IL-10 levels, other mechanisms could explain the anti-inflammatory activities exerted by *C. verbenacea.* Fernandes et al. [39] demonstrated that its essential oil is able to inhibit the production of prostaglandin-E2 (PGE2) and regulate inflammatory proteins, such as COX-2 and inducible nitric oxide synthase (iNOS) enzymes. Contradictorily, Passos et al. [27] showed that the anti-inflammatory action of *C. verbenacea* was not related to the decline of PGE2 levels, suggesting that the mechanism of action of this natural plant seems to be different from that of non-steroidal anti-inflammatories. Obviously, since the host-defense mechanisms are sustained by an enormous network of pro- and anti-inflammatory mediators that may exert antagonist and/or synergic biological activities, further studies are required to better characterize the role of *C. verbenacea* in the modulation of immune-inflammatory responses when periodontitis is present.

Previous findings have also indicated that constituents of *C. verbenacea*, such as the sesquiterpenes α-humulene, that exhibit a rapid onset and good absorption following topical administration are probably responsible for the anti-inflammatory actions displayed by the essential oil of this natural plant [27,31]. Accordingly, the purity of α-humulene in the *C. verbenacea* essential oil used in the present experiments was 99%, supporting the positive impact of this component in the anti-inflammatory effect of this medicinal plant.

In association with the better knowledge of the role of host immune response in modulating periodontal collapse, investigators have focused on the advance of novel therapeutic strategies of host-modulatory agents for the treatment of chronic inflammatory diseases, including periodontal disorders [5,43,44]. NSAIDs are considered an important pharmacologic class of agents that act as modulators of the host response, modifying the progression of periodontal disease, as evidenced by animal studies [10,11,15]. Although some evidences are controversial, clinical data have also shown that the systemic administration of these drugs may offer supplementary benefits in periodontal therapy when combined with scaling and root planning [12-14]. However, the systemic use of both non-selective and selective inhibitors of COX-2 are frequently linked to various side effects, including gastroduodenal and renal complications, impairing patient compliance to their use, especially when continued periods of administration are required [16]. The topical administration of these and other anti-inflammatory agents seems also to not promote satisfactory effects in modulating host immune response [17-19].

Conversely, earlier data have demonstrated that the natural agent *C. verbenacea* displayed an anti-inflammatory activity linked to a vital protective effect on the gastric mucosa, and very low toxicity in acute models of experimentation in rats when systemically or topically administered [27-31]. Moreover, it was revealed that *C. verbenacea* presents an imperative antiulcer effect, contributing to the preservation of mucosal integrity [45]. It is important to mention that previous findings demonstrated that the potent anti-inflammatory activity of this medicinal plant has an efficacy similar to that of well-established NSAIDs [30]. For that reason, the use *C. verbenacea* would be important strategy to modulate periodontal disease, avoiding the adverse effects attributed to other anti-inflammatory drugs, especially we consider that their positive effects may be achieved using local administration, as observed in this study. Further, this therapeutic alternative would also allow the possibility of a longer period of drug administration in periodontal disease treatment.

In addition to these exposed advantages concerning the topical use of *C. verbenacea* essential oil*,* evidence demonstrating the antibacterial potential of *C. verbenacea* and its components [31-33] could greatly support their utilization in the therapy of infectious diseases such as periodontitis. Our microbiological data demonstrated that this medicinal plant might positively interfere in the decrease of putative periodontopathogens. While a trend toward the reduction of *A. actinomycetemcomitans* has been observed, a significant decline of *P. gingivalis* was verified in the present microbiological assays. In line with these findings, Hernardez et al. [46] demonstrated the antibacterial activity of the essential oil of *C. verbenacea* against bacteria related to gastrointestinal, respiratory, and dermatological disorders, both Gram-negative and Gram-positive. On the other hand, Michielin et al. [33] revealed that some extracts from *C. verbenacea* were more effective against Gram-positive (*Staphylococcus aureus* and *Bacillus cereus*) than Gram-negative bacteria (*Escherichia coli* and *Pseudomonas aeruginosa*). Of interest, to our knowledge, this is the first study to investigate the antibacterial impact of topically applied *C. verbenacea* in pathogens related to periodontitis, hampering a more direct comparison with the outcomes from other investigations. Although further investigations are needed to determine the exact mechanisms involved in the antibacterial effect of *C. verbenacea*, the antimicrobial activity displayed by the essential oil in extracts of this plant can be attributed to the presence of constituents such as aromatic compounds, which have previously shown antimicrobial activity [33]. Nevertheless, additional investigations are required to establish which constituents are accountable for their antibacterial properties. Interestingly, *T. forsythia* was not found in any ligature biofilm in both groups of the present study. In fact, although this species is considered a recognized periodontal pathogen associated with human subgingival biofilm in periodontal pockets, other studies assessing the ligature-induced biofilm in rats also did not find *T. forsythia* among the bacteria population [47].

Within the limitations of this investigation, it was demonstrated that *C. verbenacea* oil topical preparation was effective in protecting alveolar bone loss in ligature-induced periodontitis, which might be mediated, in part, by its inhibitory effect on the periodontal pathogens and, in part, by its modulatory role in the immune-inflammatory response. In the current paradigm of periodontal disease, periodontal pathogens are required for disease beginning; however, the extent and severity of periodontal destruction are dependent on the nature of host response to the bacterial challenge. Until now, most of the therapeutic agents suggested as adjunctive to periodontal therapy targeted antibiotic or anti-inflammatory effects. The development of novel therapeutic strategies combining simultaneously both the host-modulatory effect and the antibacterial activity would increase the likelihood of successfully managing periodontitis. The attractive outcomes observed in the present study, linked with the combined anti-inflammatory and antibacterial therapeutic actions of *C. verbenacea*, support the continued investigation of this plant as a potential new way to control the deterioration of tooth-supporting tissues in individuals suffering from periodontal disease.

It is important to highlight that the present study did not include therapeutic groups to evaluate other anti-inflammatory agent effects since the primary aim of this investigation was to assess the effects of *C. verbenacea*. As this study was the first to analyze whether the topical application of *C. verbenacea* could promote some protective effect against periodontal destruction, only a control group using a vehicle substance was used in comparison with the *C. verbenacea* essential oil group. Although it may be considered a limitation of the present trial, we believe that the absence of other comparative groups – such as saline or another anti-inflammatory agent group – does not invalidate the outcomes obtained in the present investigation*.* Nevertheless, a comparison with other therapies could bring additional information concerning the real relevance of *C. verbenacea* in modulating periodontitis, and it needs to be evaluated in future investigations.

Recently, the Brazilian market released this plant for the industrial production of therapeutic agents, and a commercial preparation with *C. Verbenacea* is available to treat musculo-skeletal disorders and tendinitis. Further data are required to consider this natural agent and its constituents as adjunctive in periodontal therapy in clinical practice, and provide new insights for its antibacterial action and its modulation of periodontal disease progression.

## Conclusion

*C. verbenacea* oil topical preparation is effective in protecting alveolar bone loss in ligature-induced periodontitis, which might be mediated, in part, by its inhibitory effect on the periodontal pathogens and, in part, by its modulatory role in the immune-inflammatory response.

## Competing interests

There is no conflict of interest to declare.

## Authors’ contributions

SPP participated in the conception and design of study and helped to induce experimental periodontitis / GEB carried out the measurement of alveolar bone loss / RCVC carried out the ELISA assays and microbiological assessment / FRC helped to induce experimental periodontitis and performed the statistical analysis / MZC helped to morphometric assay, participated in analysis and interpretation of data and in the drafted the manuscript / MAF and GMF carried out chemical analyses related to plant material and extraction of essential oil / FVR participated in the design of study and in the drafted the manuscript and carried out the ELISA assays / All authors read and approved the final manuscript.

## Pre-publication history

The pre-publication history for this paper can be accessed here:

http://www.biomedcentral.com/1472-6882/12/224/prepub

## References

[B1] Tonetti MS Claffey N European Workshop in Periodontology group C Advances in the progression of periodontitis and proposal of definitions of a periodontitis case and disease progression for use in risk factor research. Group C consensus report of the 5th European Workshop in Periodontology J Clin Periodontol 2005 32 Suppl 6 210 213 16128839 10.1111/j.1600-051X.2005.00822.x

[B2] Burt B Position paper: epidemiology of periodontal diseases J Periodontol 2005 76 1406 1419 16101377 10.1902/jop.2005.76.8.1406

[B3] Kinane DF Preshaw PM Loos BG Working Group 2 of Seventh European Workshop on Periodontology Host-response: understanding the cellular and molecular mechanisms of host-microbial interactions-consensus of the Seventh European Workshop on Periodontology J Clin Periodontol 2011 38 Suppl 11 44 48 21323703 10.1111/j.1600-051X.2010.01682.x

[B4] Reinhardt RA Stoner JA Golub LM Association of gingival crevicular fluid biomarkers during periodontal maintenance with subsequent progressive periodontitis J Periodontol 2010 8 251 259 20151804 10.1902/jop.2009.090374 PMC2822998

[B5] Guimarães MR Coimbra LS De Aquino SG Spolidorio LC Kirkwood KL Rossa C Jr Potent anti-inflammatory effects of systemically administered curcumin modulate periodontal disease in vivo J Periodontal Res 2011 46 269 279 10.1111/j.1600-0765.2010.01342.x 21306385 PMC3086370

[B6] Vieira Ribeiro F De Mendonça AC Santos VR Bastos MF Figueiredo LC Duarte PM Cytokines and bone-related factors in systemically healthy patients with chronic periodontitis and patients with type 2 diabetes and chronic periodontitis J Periodontol 2011 82 1187 1196 10.1902/jop.2011.100643 21284550

[B7] Graves D Cytokines that promote periodontal tissue destruction J Periodontol 2008 79 1585S 1591S 10.1902/jop.2008.080183 18673014

[B8] Bloemen V Schoenmaker T de Vries TJ Everts V IL-1β favors osteoclastogenesis via supporting human periodontal ligament fibroblasts J Cell Biochem 2011 112 1890 1897 10.1002/jcb.23109 21433061

[B9] Sasaki H Okamatsu Y Kawai T Kent R Taubman M Stashenko P The interleukin-10 knockout mouse is highly susceptible to Porphyromonas gingivalis-induced alveolar bone loss J Periodontal Res 2004 432 441 441 10.1111/j.1600-0765.2004.00760.x 15491348

[B10] Gurgel BC Duarte PM Nociti FH JR Impact of an anti-inflammatory therapy and its withdrawal on the progression of experimental periodontitis in rats J Periodontol 2004 75 1613 1618 10.1902/jop.2004.75.12.1613 15732862

[B11] Holzhausen M Spolidorio DM Muscará MN Hebling J Spolidorio LC Protective effects of etoricoxib, a selective inhibitor of cyclooxygenase-2, in experimental periodontitis in rats J Periodontal Res 2005 40 208 211 10.1111/j.1600-0765.2005.00787.x 15853965

[B12] Sekino S Ramberg P Lindhe J The effect of systemic administration of ibuprofen in the experimental gingivitis model J Clin Peridontol 2005 32 182 187 10.1111/j.1600-051X.2005.00671.x 15691349

[B13] Aras H Cağlayan F Güncü GN Berberoğlu A Kilinç K Effect of systemically administered naproxen sodium on clinical parameters and myeloperoxidase and elastase-like activity levels in gingival crevicular fluid J Periodontol 2007 78 868 873 10.1902/jop.2007.060412 17470020

[B14] Azoubel MC Sarmento VA Cangussú V Adjunctive benefits of systemic etoricoxib in non-surgical treatment of aggressive periodontitis: short-term evaluation J Periodontol 2008 79 1719 1725 10.1902/jop.2008.080019 18771374

[B15] Queiroz-Junior CM Pacheco CM Maltos KL Caliari MV Duarte ID Francischi JN Role of systemic and local administration of selective inhibitors of cyclooxygenase 1 and 2 in an experimental model of periodontal disease in rats J Periodontal Res 2009 44 153 160 10.1111/j.1600-0765.2007.01069.x 19210344

[B16] Fitzgerald GA Patrono C The coxibs, selective inhibitors of cyclooxygenase-2 N Engl J Med 2001 345 433 442 10.1056/NEJM200108093450607 11496855

[B17] Kostrica R Rottenberg J Kvech J Betka J Jablonicky J Randomised, double-blind comparison of efficacy and tolerability of diclofenac mouthwash versus placebo in mucositis of oral cavity by radiotherapy J Clin Res 2002 5 1 15

[B18] Xu Y Höfling K Fimmers R Frentzen M Jervøe-Storm PM Clinical and microbiological effects of topical subgingival application of hyaluronic acid gel adjunctive to scaling and root planing in the treatment of chronic periodontitis J Periodontol 2004 75 1114 1118 10.1902/jop.2004.75.8.1114 15455740

[B19] Agarwal S Mathur S Kothiwale S Benjamin A Efficacy and acceptability of 0.074% diclofenac-containing mouthwash after periodontal surgery: a clinical study Indian J Dent Res 2010 21 408 412 10.4103/0970-9290.70814 20930354

[B20] Calixto JB Campos MM Otuki MF Santos AR Anti-inflammatory compounds of plant origin. Part II. modulation of pro-inflammatory cytokines, chemokines and adhesion molecules Planta Med 2004 70 93 103 14994184 10.1055/s-2004-815483

[B21] Paterniti I Briguglio E Mazzon E Effects of Hypericum Perforatum, in a rodent model of periodontitis BMC Complement Altern Med 2010 23 10 73 21092263 10.1186/1472-6882-10-73 PMC3000377

[B22] Ozdemir H Kara MI Erciyas K Ozer H Ay S Preventive effects of thymoquinone in a rat periodontitis model: a morphometric and histopathological study J Periodontal Res 2012 47 74 80 10.1111/j.1600-0765.2011.01406.x 21992581

[B23] Botelho MA Rao VS Montenegro D Effects of a herbal gel containing carvacrol and chalcones on alveolar bone resorption in rats on experimental periodontitis Phytother Res 2008 22 442 449 10.1002/ptr.2325 18338370

[B24] Basri DF Tan LS Shafiei Z Zin NM In vitro antibacterial activity of galls of Quercus infectoria Olivier against oral pathogens Evid Based Complement Alternat Med 2012 2012 632796 22203875 10.1155/2012/632796 PMC3235900

[B25] Toker H Ozan F Ozer H Ozdemir H Eren K Yeler H A morphometric and histopathologic evaluation of the effects of propolis on alveolar bone loss in experimental periodontitis in rats J Periodontol 2008 79 1089 1094 10.1902/jop.2008.070462 18533788

[B26] Napimoga MH Benatti BB Lima FO Cannabidiol decreases bone resorption by inhibiting RANK/RANKL expression and pro-inflammatory cytokines during experimental periodontitis in rats Int Immunopharmacol 2009 9 216 222 10.1016/j.intimp.2008.11.010 19070683

[B27] Passos GF Fernandes ES Da Cunha FM Anti-inflammatory and anti-allergic properties of the essential oil and active compounds from Cordia verbenacea J Ethnopharmacol 2007 110 323 333 10.1016/j.jep.2006.09.032 17084568

[B28] de Oliveira DM Luchini AC Seito LN Gomes JC Crespo-López ME Di Stasi LC Cordia verbenacea and secretion of mast cells in different animal species J Ethnopharmacol 2011 135 463 468 10.1016/j.jep.2011.03.046 21453767

[B29] Sertié JA Basile AC Panizza S Oshiro TT Azzolini CP Penna SC Pharmacological assay of Cordia verbenacea. III: oral and topical antiinflammatory activity and gastrotoxicity of a crude leaf extract J Ethnopharmacol 1991 31 239 247 10.1016/0378-8741(91)90008-2 2023431

[B30] Sertié JA Woisky RG Wiezel G Rodrigues M Pharmacological assay of Cordia verbenacea V: oral and topical anti inflammatory activity, analgesic effect and fetus toxicity of a crude leaf extract Phytomedicine 2005 12 338 344 10.1016/j.phymed.2003.09.013 15957367

[B31] de Carvalho PM JR Rodrigues RF Sawaya AC Marques MO Shimizu MT Chemical composition and antimicrobial activity of the essential oil of Cordiaverbenacea D.C J Ethnopharmacol 2004 95 297 301 10.1016/j.jep.2004.07.028 15507352

[B32] Meccia G Rojas LB Velasco J Chemical composition and antibacterial activity of the essential oil of Cordia verbenacea from the Venezuelan Andes Nat Prod Commun 2009 4 1119 1122 19768996

[B33] Michielin EM Salvador AA Riehl CA Smânia AJR Smânia EF Ferreira SR Chemical composition and antibacterial activity of Cordia verbenacea extracts obtained by different methods Bioresour Technol 2009 100 6615 6623 10.1016/j.biortech.2009.07.061 19683436

[B34] Fernandes MI Gaio EJ Oppermann RV Rados PV Rosing CK Comparison of histometric and morphometric analyses of bone height in ligature-induced periodontitis in rats Braz Oral Res 2007 21 216 221 17710286 10.1590/s1806-83242007000300005

[B35] Sardi JC Duque C Camargo GA Hofling JF Gonçalves RB Periodontal conditions and prevalence of putative periodontopathogens and Candida spp. in insulin-dependent type 2 diabetic and non-diabetic patients with chronic periodontitis-A pilot study Arch Oral Biol 2011 56 1098 1105 10.1016/j.archoralbio.2011.03.017 21529777

[B36] Casarin RC Ribeiro Edel P Mariano FS Nociti FH Jr Casati MZ Gonçalves RB Levels of Aggregatibacter actinomycetemcomitans, Porphyromonas gingivalis, inflammatory cytokines and species-specific immunoglobulin G in generalized aggressive and chronic periodontitis J Periodontal Res 2010 45 635 642 10.1111/j.1600-0765.2010.01278.x 20546109

[B37] Elkhouli AM The efficacy of host response modulation therapy (omega-3 plus low-dose aspirin) as an adjunctive treatment of chronic periodontitis (clinical and biochemical study) J Periodontal Res 2011 46 261 268 10.1111/j.1600-0765.2010.01336.x 21261621

[B38] Kasuyama K Tomofuji T Ekuni D Effects of topical application of inorganic polyphosphate on tissue remodeling in rat inflamed gingiva J Periodont Res 2012 47 2 159 164 10.1111/j.1600-0765.2011.01414.x 21923677

[B39] Fernandes ES Passos GF Medeiros R Anti-inflammatory effects of compounds alpha-humulene and (-)-trans-caryophyllene isolated from the essential oil of Cordia verbenacea Eur J Pharmacol 2007 569 228 236 10.1016/j.ejphar.2007.04.059 17559833

[B40] Chou WY Lu CN Lee TH Electroporative interleukin-10 gene transfer ameliorates carbon tetrachloride-induced murine liver fibrosis by MMP and TIMP modulation Acta Pharmacol Sin 2006 27 469 476 10.1111/j.1745-7254.2006.00304.x 16539848

[B41] Claudino M Trombone AP Cardoso CR The broad effects of the functional IL-10 promoter-592 polymorphism: modulation of IL-10, TIMP-3, and OPG expression and their association with periodontal disease outcome J Leukoc Biol 2008 84 1565 1573 10.1189/jlb.0308184 18725394

[B42] Claudino M Garlet TP Cardoso CR Down-regulation of expression of osteoblast and osteocyte markers in periodontal tissues associated with the spontaneous alveolar bone loss of interleukin-10 knockout mice Eur J Oral Sci 2010 118 19 28 10.1111/j.1600-0722.2009.00706.x 20156261

[B43] Feldmann M Development of anti-TNF therapy for rheumatoid arthritis Nat Rev Immunol 2002 2 364 371 10.1038/nri802 12033742

[B44] Rogers JE Li F Coatney DD A p38 mitogen-activated protein kinase inhibitor arrests active alveolar bone loss in a rat periodontitis model J Periodontol 2007 78 1992 1198 10.1902/jop.2007.070101 18062121

[B45] Roldão Ede F Witaicenis A Seito LN Hiruma-Lima CA Di Stasi LC Evaluation of the antiulcerogenic and analgesic activities of Cordia verbenacea DC. (Boraginaceae) J Ethnopharmacol 2008 119 94 98 10.1016/j.jep.2008.06.001 18588967

[B46] Hernandez T Canales M Teran B Antimicrobial activity of the essential oil and extracts of Cordia curassavica (Boraginaceae) J Ethnopharmacol 2007 111 137 141 10.1016/j.jep.2006.11.002 17140754

[B47] Duarte PM Tezolin KR Figueiredo LC Feres M Bastos MF Microbial profile of ligature-induced periodontitis in rats Arch Oral Biol 2010 55 142 147 10.1016/j.archoralbio.2009.10.006 19931851

